# Conjunctive Analyses of BSA-Seq and BSR-Seq to Reveal the Molecular Pathway of Leafy Head Formation in Chinese Cabbage

**DOI:** 10.3390/plants8120603

**Published:** 2019-12-13

**Authors:** Rui Li, Zhongle Hou, Liwei Gao, Dong Xiao, Xilin Hou, Changwei Zhang, Jiyong Yan, Lixiao Song

**Affiliations:** 1 State Key Laboratory of Crop Genetics and Germplasm Enhancement, and Key laboratory of Biology and Genetic Improvement of Horticultural Crops, Ministry of Agriculture, College of Horticulture, Nanjing Agricultural University, Nanjing 210095, China; 2017104078@njau.edu.cn (R.L.); 2018804231@njau.edu.cn (Z.H.); 2016204018@njau.edu.cn (L.G.); dong.xiao@njau.edu.cn (D.X.); hxl@njau.edu.cn (X.H.); 2 Jiangsu Key Laboratory for Horticultural Crop Genetic Improvement, Jiangsu Academy of Agricultural Sciences, Nanjing 210014, China; js2018slx@163.com

**Keywords:** Chinese cabbage, leafy head formation, BSA-Seq, BSR-Seq, phytohormones, calcium signaling pathway, *BrCKL8*

## Abstract

As the storage organ of Chinese cabbage, the leafy head was harvested as a commercial product due to its edible value. In this study, the bulked segregant analysis (BSA) and bulked segregant RNA-Seq (BSR) were performed with F_2_ separation progeny to study the molecular mechanism of leafy head formation in Chinese cabbage. BSA-Seq analysis located four candidate regions containing 40 candidate genes, while BSR-Seq analysis revealed eight candidate regions containing 607 candidate genes. The conjunctive analyses of these two methods identified that Casein kinase gene *BrCKL8* (*Bra035974*) is the common candidate gene related with leafy head formation in Chinese cabbage, and it showed high expression levels at the three segments of heading type plant leaves. The differentially expressed genes (DEGs) between two set pairs of cDNA sequencing bulks were divided into two categories: one category was related with five hormone pathways (Auxin, Ethylene, Abscisic acid, Jasmonic acid and Gibberellin), the other category was composed of genes that associate with the calcium signaling pathway. Moreover, a series of upregulated transcriptional factors (TFs) were also identified by the association analysis of BSR-Seq analysis. The leafy head development was regulated by various biological processes and effected by diverse external environment factors, so our research will contribute to the breeding of perfect leaf-heading types of Chinese cabbage.

## 1. Introduction

Chinese cabbage (*Brassica rapa* ssp. *pekinensis*) originates in China, is one of the widely cultivated horticultural crops in Asia [1]. The vegetative growth of Chinese cabbage is divided into four growth stages, i.e., seeding, rosette, folding and heading stages [2]. After folding stage, the newly formed leaves begin to bold and curl inward at the top, which are essential for tight leafy head formation. Leafy head tightness and size are important indices to assessing the economic value of Chinese cabbage [3]. However, the molecular mechanism of leafy head formation in Chinese cabbage remains elusive. The leafy head development of Chinese cabbage is affected by diversity factors, such as phytohormones’ uneven distribution [4], low temperature induction [5] and leafy shape [6].

The genetical genomics approach identified that four phytohormones (Cytokinin, Auxin, Gibberellins and Jasmonic acid) function to regulate the development of leafy head [7]. Gu et al. also demonstrated that the IAA and ABA play important roles in heading type formation [8]. As plant growth phytohormone, auxin plays critical roles in many plant developmental processes [9]. Previously, transgenic Chinese cabbage with an auxin biosynthesis gene showed more rapid and larger size head formation [2]. Gao et al. (2017) reported that influx auxin carrier BrAUX/LAX and efflux auxin carrier BrPIN and BrPGP played critical roles in Chinese cabbage leafy head formation by regulating auxin uneven distribution [4].

The orientation of leaf curvature was effected by the antagonism of adaxial and abaxial polarity genes, where the inward-curling leaves converge to form the leafy head of Chinese cabbage [10]. For the construction of leaf morphology, many genes and environmental factors were involved in this process [11]; the upward or downward curvature of leaves was correlated with the changes in the ratios of leaf length to width and leaf length to petiole length [12]. In *Arabidopsis*, the transcriptional level of HYL1 in leaves was related to the extent of leaf curvature [12]. Along with the fact that two orthologs of *Arabidopsis BREVIS RADIX* (*BRX*) gene (*BrBRX.1* and *BrBRX.2*) were identified as candidate genes that associated with leafy head formation in *Brassica rapa*, other four leaf adaxial genes have also been identified to involve in this process, including paralogous pair of auxin response factor *BrARF3.1* and *BrARF4.1*, the GARP family gene *BrKAN2.1* and *BrKAN2.3* [7]. Additionally, some special genes were also involved in the leaf heading process, *BrpSPL9-2*-overexpressing transgenic plants showed earliness of heading time [13]. The uneven distribution of BrpTCP transcription in heading leaves can lead to the conversion of leafy head shape in Chinese cabbage [6]. The gene silencing of *BrAN3* stimulated the leafy head formation and modulated multiple hormone signaling pathways (auxin, ethylene, GA, JA, ABA, BR, CK and SA) [14].

In our present study, we obtained the F_2_ hybridization between two Chinese cabbage types, heading type cultivar W30 (male) and non-heading type cultivar 082 (female). BSA-Seq and BSR-Seq analyses were used to reveal the critical pathway of Chinese cabbage leafy head formation. Global gene expression pattern analysis showed that a large percentage of DEGs were enriched to five plant hormone signaling pathways (Auxin, Ethylene, Abscisic acid, Jasmonic acid and Gibberellin) and the calcium signaling pathway. Based on BSR-Seq, several transcription factors, such as WRKY, MYB and bZIP were also identified by SNP-index analysis. Our real time PCR result showed that the expression levels of Casein kinase gene *BrCKL8* (*Bra035974*), auxin biosynthesis gene *BrYUCCA5* (*Bra036002*) and calcium signaling-related gene *BrCBL9* (*Bra022104*) were higher in the three parts of mature leaves of heading type plants than non-heading type plants. Our result will promote us to grasp the changes of transcriptional profiling of Chinese cabbage leafy head formation and could be a valuable resource for the breeder to developing desirable Chinese cabbage cultivars.

## 2. Results

### 2.1. Bulked-Segregant Analysis

#### 2.1.1. Sequencing Data Analysis of Four DNA Bulks

BSA-seq analysis was performed with the DNAs of four libraries (R01–R04) using the Illumina HiSeq™2500 platform (Illumina, San Diego, CA, USA). In total, 287.61 million paired-end reads were generated, approximately 37.95 million and 36.84 million reads for R01 and R02, while 107.06 million and 105.76 million reads originated from R03 and R04, respectively. After mapping of the clean reads to the reference Chinese cabbage genome (http://brassicadb.org/brad/), resulting in 27× coverage for parental bulks and 75× coverage for two F_2_ progeny bulks (Table 1), the mapping quality was over 92.44% and the alignment efficiency reached to at least 96.71%. In addition, the insert size generally conformed to the normal distribution.

#### 2.1.2. Association Analysis of BSA-Seq Data

**Figure 1:**
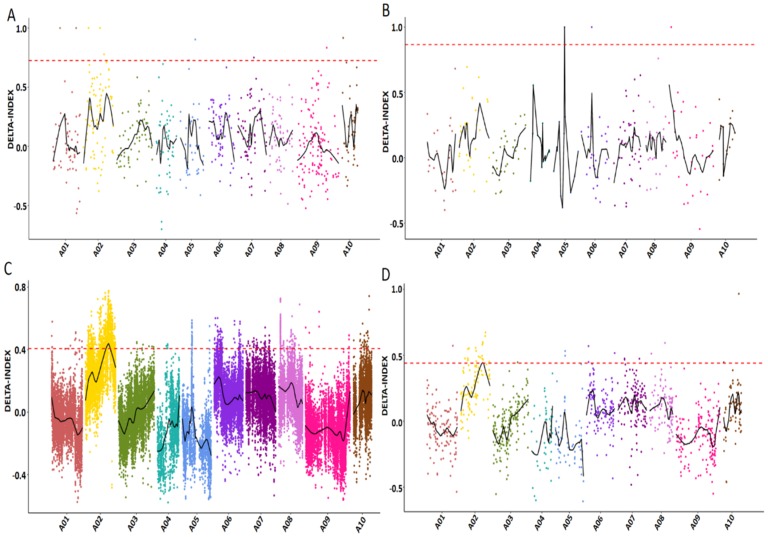
The calculation of Δ(SNP-index) values on chromosomes to identify the candidate regions that associated with the leaf heading trait in Chinese cabbage, based on BSA-Seq and BSR-Seq analysis. The abscissa indicates the position of the chromosome, the ordinate indicates the Δ(SNP-index) values. The scatter point represents the Δ(SNP-index) values calculated for specific SNP or small indels. The curved black lines are average Δ(SNP-index) values. The red dashed line represents the threshold line for the Loess regression (p-value ≤ 0.01). (**A**) Candidate regions were located by the association analysis of specific SNPs between the two segregate bulks of BSA-Seq (R03 and R04); (**B**) Candidate regions were located by the association analysis of specific small indels between R03 and R04; (**C**) Candidate regions were located by the association analysis of specific SNPs between the two segregate bulks of BSR-Seq (T03 and T04); (**D**) Candidate regions were located by the association analysis of specific small indels between T03 and T04.

199,901 SNPs and 51,429 small indels were identified between two F_2_ sequencing bulks (R03 and R04) by using the GATK toolkit [15], then 3841 SNPs and 1133 small indels with different genotypes between the two samples were selected for association analysis (coverage depth > 5×). Four candidate regions responsible for leafy head formation were located from peaks with an Δ(SNP-index) above the threshold of 99% (Figure 1A,B), which includes 40 candidate genes (Table 2). Pathway definitions were derived from the KOG (euKaryotic Ortholog Groups) database, which showed that a proportion of candidate genes were enriched to nine pathways, including signal transduction mechanisms, coenzyme transport and metabolism, energy production and conversion, posttranslational modification, protein turnover, chaperones, et al. (Figure S1), indicating that the leafy head development may associate with signaling transduction and various biochemical processes. Among these candidate genes, we identified one Casein kinase gene *BrCKL8* (*Bra035974*) in Scaffold000111, and it has reported that the *Arabidopsis Casein kinase 1-like 8* (*CKL8*) gene was involved in the regulation of ethylene biosynthesis. Additionally, there are another two representative genes, one *ABF/AREB* family gene *BrABF2* (*Bra040260*) was identified to associate with the ABA signaling pathway and located in Scaffold000193, one *KANADI* family gene *BrKAN2* (*Bra039528*) was identified to involve in the regulation of plant adaxial–abaxial polarity and located in Scaffold000167.

### 2.2. Bulked Segregant RNA-Seq Analysis

#### 2.2.1. Sequencing Data Analysis of Four cDNA Bulks

To gain insight into the transcriptional profiling of leafy head formation in Chinese cabbage, the bulked segregant RNA-seq (BSR-Seq) analysis was performed with four cDNA sequencing bulks (T01–T04) by using the Illumina HiSeq™2500 platform (Illumina, San Diego, CA, USA). The parent bulks (T01 and T02) generated 21.75 million and 17.80 million clean reads, the two F_2_ segregation bulks (T03 and T04) generated 62.66 million and 52.15 million clean reads, respectively; Q30 of four cDNA sequencing bulks were over 91.87%. Clean reads were aligned to the reference Chinese cabbage genome (http://brassicadb.org/brad/), the alignment efficiency reached to 87.56% at least (Table 3).

**Figure 2:**
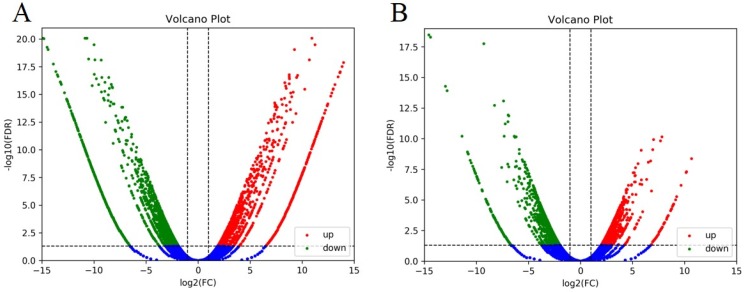
Volcano plots of differentially-expressed genes (DEGs) between two set pairs of cDNA sequencing bulks of BSR-Seq analysis, based on log_2_FC and FDR values. Green scatter points represent the upregulated genes, red scatter points represent the downregulated genes. (**A**) T01 vs. T02. (**B**) T03 vs. T04.

There are 3673 genes with differential expression pattern (DEGs) between parent bulks (T01 vs. T02), with 1068 genes significantly upregulated and 2065 significantly downregulated (Figure 2A). A total of 2517 DEGs were identified between T03 and T04 bulks, which includes 995 upregulated genes and 1522 down-regulated genes (Figure 2B).

#### 2.2.2. Comparison of Gene Expression Files between Two Set Pairs of cDNA Sequencing Bulks

**Figure 3:**
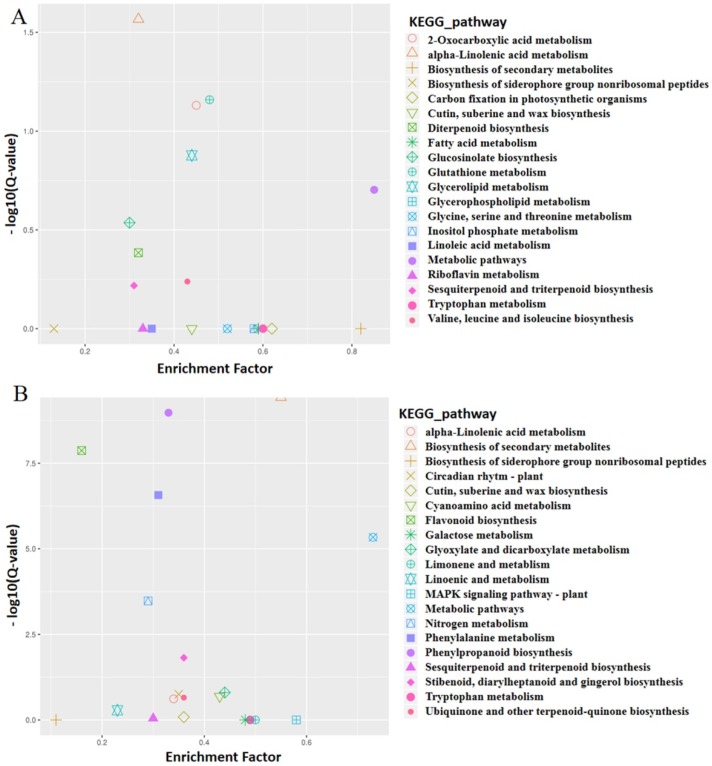
Graph of KEGG pathways analysis of differentially expressed genes (DEGs) between two set pairs of cDNA sequencing bulks. (**A**) T01 vs. T02. (**B**) T03 vs. T04.

To further explore the key genes involved in the regulation of leafy head formation, the DEGs between two set pairs of cDNA sequencing bulks (T01 vs. T02, T03 vs. T04) were annotated to the GO (Gene Ontology), COG (Cluster of Orthologous Groups of proteins), KOG (euKaryotic Ortholog Groups), KEGG (Kyoto Encyclopedia of Genes and Genomes) public databases. As a result, DEGs from four cDNA sequencing bulks (T01–T04) and all genes were assigned to three major GO terms, where a big proportion of DEGs were identified in biological processes and enriched in 17 subclusters (Figure S2). For the two parent bulks (T01 and T02), a total of 536 DEGs were assigned to 124 KEGG pathways, the number of genes associated with each pathway ranged from 1 to 208, where one pathway was significantly enriched (*p*-value ≤ 0.05), that being the alpha-linolenic acid metabolism (Figure 3A). For the T03 and T04 bulks, a total of 438 DEGs were assigned to 120 KEGG pathways, and the number of genes associated with each pathway ranged from 1 to 197. There are seven pathways were significantly enriched (*p*-value ≤ 0.05): biosynthesis of secondary metabolites, phenylpropanoid biosynthesis, flavonoid biosynthesis, phenylalanine metabolism, metabolic pathways, nitrogen metabolism, stilbenoid, diarylheptanoid and gingerol biosynthesis (Figure 3B). These results indicated that the leafy head development may associate with various metabolism and biological process.

The KOG function classification analysis showed that the DEGs were enriched to 23 pathways including amino acid transport and metabolism, the posttranslational modification, protein turnover, chaperones and signal transduction mechanisms et al. (Figure S3). Firstly, the proportion of phytohormone genes with a differently expressed pattern were identified between T03 and T04 bulk, a total of 21 DEGs assigned to the auxin pathway showed that one auxin efflux gene *BrPIN5* was upregulated and two orthologs of the auxin biosysthetic gene *YUCCA8* were downregulated. As early auxin responsive genes, the transcript levels of three homologs of the *BrGH3* gene were reduced; three orthologs of *Arabidopsis SMALL AUXIN UP-RNA* (*SAURs*) genes were upregulated, including *SAUR34*, *SAUR72* and *SAUR46*, while *SAUR10*, *SAUR9*, *SAUR14* and *SAUR51* were downregulated. Three orthologs of *AUXIN/INDOLEACETICACID-INDUCED PROTEINs* (*Aux/IAAs*) genes were upregulated, including *IAA30*, *IAA15* and *IAA11*, while *IAA3*, *IAA5* and *IAA29* were downregulated. Two AUXIN RESPONSE FACTOR family genes were downregulated, including *BrARF10* and *BrARF11*. The DEGs assigned to the ethylene pathway showed that the expression levels of two paralogs of the ETHYLENE RESPONSE FACTOR family gene were upregulated, including *ERF4* and *ERF5*, two homologs of *BrERF5* gene were downregulated and one negative regulator of the ethylene-activated signaling pathway *ETHYLENE RESPONSE 2* (*ETR2*) was also downregulated. The expression level of seven DEGs involved in the abscisic acid (ABA) signaling pathway were downregulated, including five *BrPYR/PYLs*, one *BrPP2C* and one *BrABF1*. Two genes related to the JA pathway were upregulated, including *BrJAR1* and *BrJMT*. The expression pattern of ten gibberellin-related genes were altered, including three *BrGASAs*, four *BrGA2oxs* and three *BrGA20oxs*. Secondly, three calcium signaling related genes were identified from the DEGs of two F_2_ segregation bulks (T03 and T04), including two calmodulin-dependent protein kinases genes (*BrCPK34* and *BrCPK12*) and one calmodulin-like gene (*BrCML38*). The details were shown in Table S1.

When T01 was compared to T02, the expression patterns of 21 DEGs related to the auxin pathway (including one *BrARF11*, one *BrIAA14*, three *BrYUCCAs*, twelve *BrSAURs* and four *BrGH3* family genes), six DEGs related to the ethylene pathway (including three homologous gene of *BrERF4*, two homologous gene of *BrERF5*, one *BrERF2*), five DEGs related to the abscisic acid (ABA) pathway (including four *BrPYR/PYLs* and one *BrPP2C*), four DEGs related to the jasmonic acid (JA) pathway (including *BrJAZ5*, *BrJAZ10*, *BrJAZ5* and *BrJMT*), and finally, six DEGs involved in gibberellin (GA) signal transduction (including one *BrGASA1*, one *BrGA20ox3*, three *BrGA2oxs* and one *BrGA3ox2*) were altered. Additionally, two calmodulin-dependent protein kinases gene (*BrCPK28* and *BrCPK10*) were also identified. The details were shown in Table S1.

#### 2.2.3. Association Analysis of BSR-Seq Data

**Figure 4:**
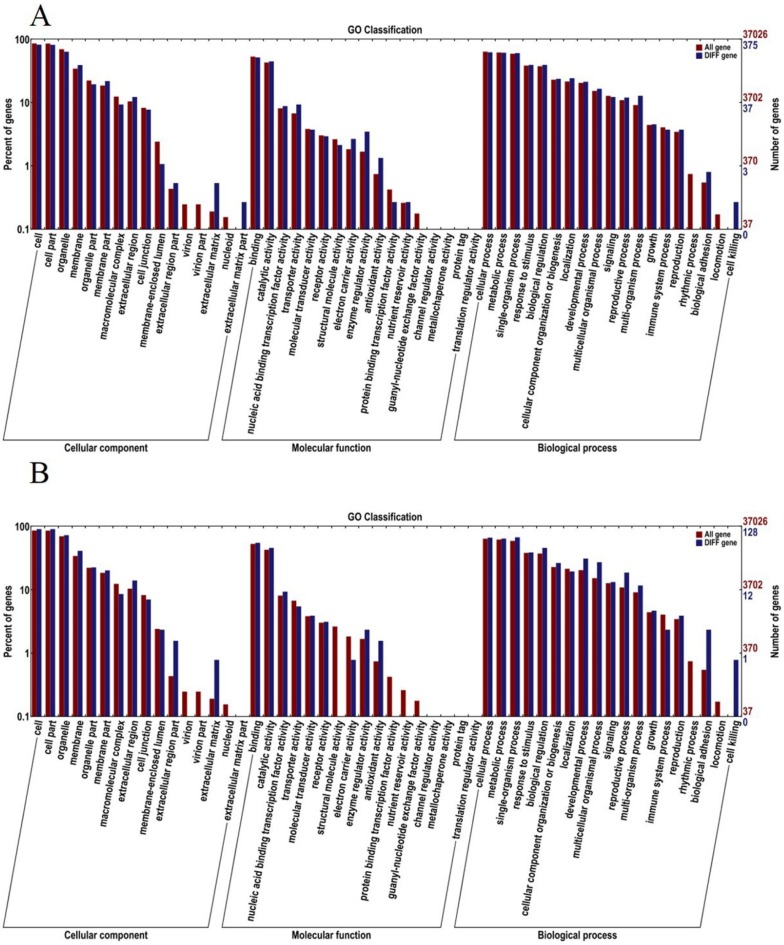
Gene Ontology (GO) enrichment analysis of candidate genes identified by association analysis of BSR-Seq analysis. All genes were assigned to the three GO categories: biological processes, cell components, and molecular functions. (**A**) Candidate genes were identified by the association analysis of specific SNPs between the two F_2_ segregate bulks of BSR-Seq (T03 and T04); (**B**) Candidate genes were identified by the association analysis of specific small indels between T03 and T04.

100,348 SNPs and 3314 INDELs with different genotypes (coverage depth > 5X) between T03 and T04 bulks were selected for association analysis. Eight candidate regions were located with a statistically remarkable peak, and six regions were identified by association analysis of specific SNPs including 456 genes (Figure 1C); the other two regions were identified by association analysis of specific small indels including 151 genes (Figure 1D). A total of 542 candidate genes were located in chromosome A02 with 5.01M length, and the details are listed in Table 4. The GO classification analysis was carried out to understand the functions of all candidate genes identified by the association analysis of SNP and small indels, which showed that in the molecular function category, the top four enriched items were binging, catalytic activity, nucleic acid binding transcription factor (TF) activity and transporter activity, while in the biological process category, the top five enriched items were cellular process, metabolic process, single-organism process, response to stimulus and biological regulation (Figure 4A,B). The KOG function classification analysis showed that these candidate genes were enriched to 21 pathways including amino acid transport and metabolism, the posttranslational modification, protein turnover, chaperones and signal transduction mechanisms et al. (Figure S4).

These results suggested that the leafy head development may associate with the various biochemical processes and signaling transduction, which was consisted with the BSA-Seq analysis result.

Among the candidate genes identified by BSA-Seq, the *BrCKL8* (*Bra035974*) gene was also identified by the SNP-index association analysis of BSR-Seq, and the crosstalk analysis of these two methods indicated that the *BrCKL8* gene was the common candidate gene related to leafy head formation in Chinese cabbage. In the plant hormone signaling pathway, two candidate genes were assigned to the ethylene pathway including one *BrERF2* (*Bra022115*) and one *BrERF9* (*Bra036016*), and in addition, four candidate genes were assigned to the auxin pathway including one auxin biosynthesis gene *BrYUCCA5* (*Bra036002*), one auxin efflux gene *BrPIN4* (*Bra026669*) and two *BrSAURs* genes (*Bra026596*, *Bra026597*). In the calcium signaling pathway, one calcineurin B-like protein (CBL) family gene *BrCBL9* (*Bra022104*) was identified in chromosome A02. Additionally, a number of transcriptional factors (TFs) were also identified in candidate regions, like the WRKY family gene *BrWRKY3* (*Bra026554*) and MYB family gene *BrMYB88* (*Bra026578*), MADS-box family gene *BrSEP4* (*Bra026543*), zf-HD family gene *BrHB21* (*Bra026589*), NAC family gene *BrNAC035* (*Bra026595*) and the bZIP family gene *BrbZIP48* (*Bra026523*).

### 2.3. Validation of Quantitative Real Time PCR for Key Genes Associated with Leafy Head Formation in Chinese Cabbage

The real time PCR was performed to identify the expression patterns of candidate genes in five individual plants, except for the fact that the Casein kinase gene *BrCKL8* (*Bra035974*), one auxin biosynthesis gene *BrYUCCA5* (*Bra036002*) and one calcium signaling-related gene *BrCBL9* (*Bra022104*) were selected from candidate regions identified by BSR-Seq.

**Figure 5:**
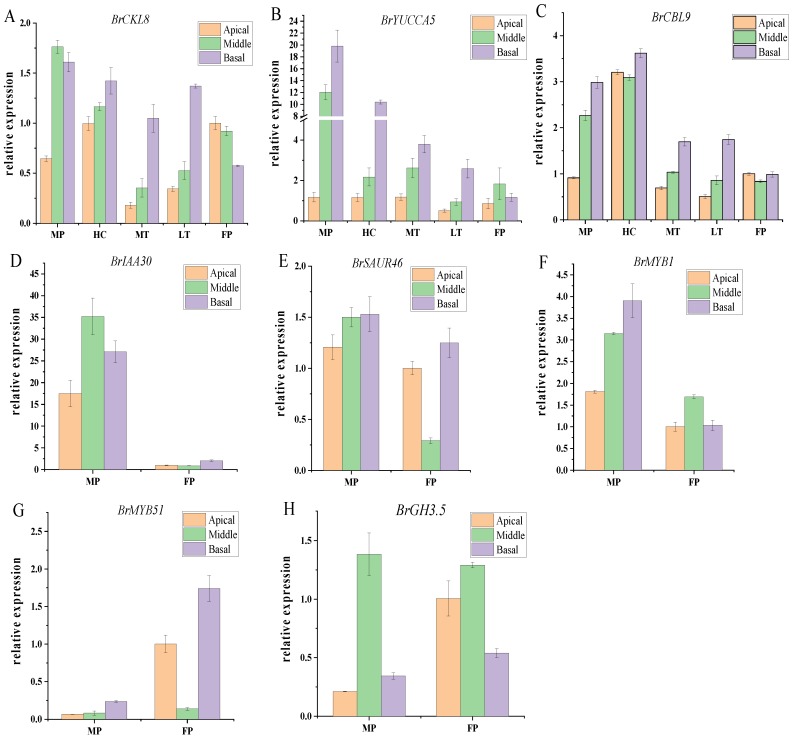
RT-qPCR validation of the expression of BSA-Seq and BSR-Seq based gene. (**A**–**C**) The relative expression levels of three specific genes *BrCKL8* (*Bra035974*), *BrYUCCA5* (*Bra036002*) and *BrCBL9* (*Bra022104*) at the three different leaf sections (apical, lateral, basal) of five individual plants. (**D**–**H**) The relative expression levels of five randomly selected genes *BrMYB51* (*Bra016553*), *BrSAUR46* (*Bra017211*), *BrMYB1* (*Bra022127*), *BrGH3.5* (*Bra026365*) and *BrIAA30* (*Bra007661*) at the three different leaf sections (apical, lateral, basal) of male parent (W30) and female parent (082). The *BrAct* (*Bra028615*) gene was used as an internal control. The transcription level at the apical of the female parent (082) leaves was set as a value of 1.0. Error bars represent the mean ± SD of three biological replicates. FP: Female parent (082); MP: Male parent (W30); HC: Leaf heading type plant; MT: Individual plant with intermediate heading type between HC and LT; LT: Non-heading type plant.

Real time PCR analysis results showed that the expression level of *BrCKL8* (*Bra035974*) was markedly higher at the three segments of male parent (W30) and outstanding heading type plant (HC) leaves comparing with the other three individual plants (Figure 5A). The expression level of *BrYUCCA5* (*Bra036002*) was significantly higher at the middle and basal part of male parent (W30) leaves, which also showed higher expression level at the basal part of outstanding heading type plant (HC) leaves (Figure 5B). The *BrCBL9* (*Bra022104*) gene showed the high expression level at the three parts of outstanding heading type plant (HC) leaves and the middle and basal parts of male parent (W30) leaves (Figure 5C). In addition, we also investigated the expression pattern of five randomly selected genes in the male and female parent, where the results were consisted with the sequencing data (Table S2), with *BrSAUR46* (*Bra017211*)*, BrMYB1* (*Bra022127*) and *BrIAA30* (*Bra007661*) being upregulated, and *BrMYB51* (*Bra016553*) and *BrGH3.5* (*Bra026365*) downregulated, which demonstrated the accuracy of the sequencing data (Figure 5D–H).

## 3. Discussion

As an agronomically quantitative trait, the leafy head formation was controlled by multiple genes, each with a small effect. Genome-wide association studies (GWAS) based on linkage disequilibrium (LD) provide a powerful tool for mapping complex traits in family-based populations [16,17], and quantitative trait locus (QTL) mapping is a traditional approach for the understanding of the genetic basis of mammal health and agronomically quantitative traits [18]. However, since it requires the development of suitable DNA markers for linkage analysis, QTL analysis has been labor-intensive and inefficient [19]. For rapidly investigating the genetic control of agronomic traits, bulked segregant analysis (BSA) and bulked segregant RNA-Seq (BSR) were developed, which just need the construction of a segregating population [20,21]. In addition, analysis of BSR-Seq data provides information on global patterns of gene expression between two segregation bulks [21]. In this study, the conjunctive analyses of BSA-Seq and BSR-Seq was performed with the F_2_ separation progeny to study the molecular mechanism of leafy head formation in genetic and transcriptional level. A total of 12 candidate regions were located by association analysis of BSA-Seq and BSR-Seq analysis, and the candidate genes were mainly located in chromosome A02 (Table 4). All DEGs from two set pairs of cDNA sequencing bulks were divided into two subgroups, the first subgroup genes were enriched to five phytohormone pathways (Auxin, Ethylene, ABA, Jasmonic acid, Gibberellin), the second subgroup of DEGs were involved in the calcium signaling pathway. Additionally, the association analysis of BSR-Seq indicated that several main transcription factors were also involved in the regulation of leafy head formation in Chinese cabbage.

As a highly conserved Serine/Threonine protein kinases family, the Casein kinase 1 (CK1) family was mainly studied in yeast and humans [22]. Certain eukaryotic CKI isoforms have the capacity to make associations with components of several signal transduction pathways [22,23] and play crucial roles in regulating the growth and development process via phosphorylating various substrates [24,25]. A rice Casein kinase 1 (OsCKL1) was identified to involve in the regulation of root development and plant hormone signaling pathways [26]. Based on the conjunctive analyses of BSA-Seq and BSR-Seq, the *BrCKL8* (*Bra035974*) gene was identified as a common candidate gene associated with leafy head formation in Chinese cabbage, where its expression level was higher at the three parts of mature leaf in heading type plants than in non-heading type plants (Figure 5A). In *Arabidopsis*, Casein kinase 1 (CK1) isoform CK1.8 negatively regulates the ethylene biosynthesis by phosphorylating the key enzyme of ethylene biosynthesis ACS5 and promoting its interaction with the E3 ubiquitin ligase Ethylene Overproduction 1 (ETO1); the *ck1.8-1* seedlings exhibited shortened hypocotyls under dark conditions and the *AtCKL8*-overexpressing transgenic plants showed an enhanced NaCl tolerance [27,28]. Ethylene takes the opposite effect on the cell expansion of petiole at the abaxial and adaxial side [29]. In this study, two ethylene-related genes were identified by association analysis of BSR-Seq, including *BrERF2* and *BrERF9*. Additionally, a total of eight ethylene DEGs were identified by BSR-Seq analysis, including four upregulated and four downregulated DEGs. Previous research has reported that the low concentration of ethylene will promote leaf elongation in sunflower (*Helianthus annuus*) and *Poa alpine* [30,31], but *Arabidopsis* overproducing the ethylene mutant are generally dwarfed [32]. Therefore, our result indicated that the dynamic balance of ethylene in heading leaves was important for the leafy head development of Chinese cabbage.

During leaf development, the upward or downward curvature of leaves was strongly associated with the establishment of adaxial-abaxial polarity in leaf primordia, which was specified by the regulation of auxin responsive factors (*ARF3* and *ARF4*) and several families of transcriptional factors, such as ARP family genes (*AS1* and *AS2*) [33,34], HD-ZIPIII family genes (PHB, PHV and REV) [35] and KANADI family genes (*KAN1* and *KAN2*) [36]. In this study, GARP transcription factor family gene *BrKAN2* (*Bra039528*), a key regulator of leaf abaxial polarity, was identified by the association analysis of BSA-Seq analysis. It has been proven that the leafy head formation in Chinese cabbage can be affected by the uneven distribution of auxin in heading leaves [4]. 

Four auxin-related genes were identified by the association analysis of BSR-Seq analysis, including *BrYUCCA5*, *BrPIN4* and two *BrSAURs* family genes. Between the two set pairs of cDNA sequencing bulks, the expression patterns of 38 auxin DEGs were altered with 13 upregulated and 26 downregulated genes, including the auxin biosynthesis gene (*YUCCA* family gene), auxin transport gene (*PIN* family gene) and the auxin responsive gene (*AUX/IAA*, *ARF*, *GH3* and *SAUR* family genes). Based on the association analysis of BSR-Seq, one auxin biosynthesis gene *BrYUCCA5* (*Bra036002*) was selected to perform real time PCR in five specific individual plants, which showed higher expression levels at the middle and basal parts of male parent leaves (W30), and at the basal parts of individual heading type plant (HC) leaves comparing with other three individual plants (Figure 5B). TFs play crucial roles during plant response to various stimuli, as well as to induce some hormonal signaling pathways to protect the plants from external stress. Moreover, a series of upregulated TFs were also identified in candidate regions, which includes the members of WRKY protein family, MYB protein family, NAC domain protein family and bZIP family. Thus, our results indicated that the auxin signaling pathway and specific transcription factors (TFs) may be involved in the regulation of leafy head formation in Chinese cabbage. 

As a second messenger in plants, calcium signals response to various external and internal stimuli, and regulate stomatal opening, root hair elongation and pollen tube growth [37,38,39]. In the calcium signaling pathway, calmodulin-like proteins (CaMs), calcium-dependent protein kinases (CDPKs) and Calcineurin B-like proteins (CBLs), were three main classes of elongation factor (EF)-hand Ca^2+^ sensors [40,41], and it has reported that the ZmCBL9 and calcineurin B-like-interacting protein kinases (ZmCIPKs) interact with each other to regulate the ABA responses in maize [42]. In rice, OsCDPK13 can be induced by cold and gibberellin in the leaf sheath, but suppressed by abscisic acid and brassinolide treatment [43]. By the association analysis of BSR-Seq, one CBL family gene *BrCBL9* (*Bra022104*) was identified as the candidate gene that was associated with leafy head formation in Chinese cabbage, and it showed a higher expression levels at the three segments of individual heading type plant (HC) leaves and the middle and basal parts of male parent leaves (W30) than other three individual plants (Figure 5C). Meanwhile, five DEGs that relate with calcium signaling were identified, including one calmodulin-like gene (*BrCML38*) and four calmodulin-dependent protein kinases genes (*BrCPK28*, *BrCPK10*, *BrCPK34* and *BrCPK12*). Among these genes, only the *BrCPK12* gene was downregulated, therefore suggesting that the calcium signaling pathway may directly participate in the regulation of leafy head formation in Chinese cabbage, and is mediated by main calcium sensors.

Phytohormones play an essential role in many aspects of plant growth and development, and help plants to adapt to diversity environmental conditions. Moreover, except the auxin- and ethylene-related genes, a total of 30 DEGs were enriched to three parallel hormone signaling pathways (ABA, Gibberellins and Jasmonates). In the ABA signaling pathway, one *ABF/AREB* family gene *BrABF2* (*Bra040260*) was identified in the candidate region by BSA-Seq analysis, where there are ten DEGs (*BrPP2C*, *BrPYR/PYLs* and *BrABF1*) with three upregulated and seven downregulated genes. Among them, the ABA responsive genes *BrABF1* have been identified as the key candidate genes that related with the leafy head morphotype of Chinese cabbage. Hilde et al. (2012) reported that enhanced gibberellin (GA) production can increase the Maize leaf elongation rate [44]. The mutants that are defective in GA biosynthesis exhibit dwarfism and a late-flowering phenotype [45,46,47]. A total of 15 DEGs were enriched to the GA signaling pathway with six upregulated and nine downregulated genes, including *BrGASAs*, *BrGA2oxs*, *BrGA20oxs* and *BrGA3ox2*. The Jasmonic acid (JA) signaling pathway was proven to be a promoter of leafy head formation in Chinese cabbage. There are five DEGs enriched to the JA signaling pathway with four upregulated genes and one downregulated gene, including *BrJAR1*, *BrJMT* and *BrJAZs*. Jasmonates (JAs) are well known to modulate plant defense and developmental processes by interacting with other phytohormones, including gibberellin, ethylene, abscisic acid and auxin [48,49,50]. Based on the crosstalk ability of various plant hormones, we suggested that these five phytohormones (Auxin, Ethylene, Abscisic acid, Jasmonic acid and Gibberellin) were ideal candidates for leafy head formation in Chinese cabbage. 

In the future, our study will focus on the specific role of plant hormones in the regulation of leafy head formation in Chinese cabbage.

## 4. Materials and Methods 

### 4.1. Plant Materials

**Figure 6:**
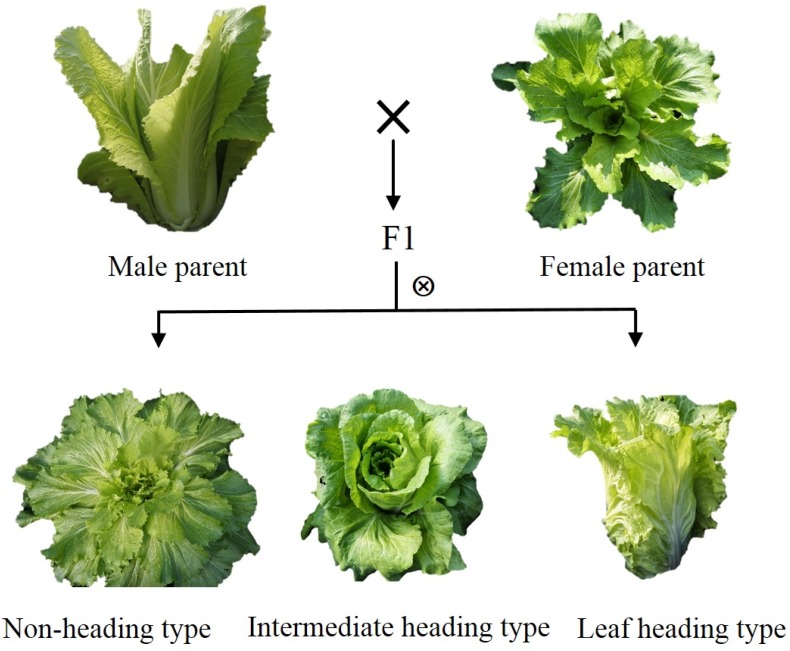
The origination of F_2_ segregation progeny with opposite leaf heading trait.

To explore the molecular mechanism of leafy head formation in Chinese cabbage, F_2_ segregation progeny was generated for high-throughput sequencing, and it was consisted with 323 F_2_ individual plants. Heading type cultivar W30 (*Brassica rapa* ssp. *pekinensis*) with leaf trichomes was crossed with non-heading type cultivar 082 (*Brassica rapa* ssp. *pekinensis*) with glabrous leaf; the former was the female parent, the latter was the male parent. The F_2_ segregation progeny was derived from the self-pollinate of outstanding F1 individuals with overlapping leaves (Figure 6), and plants were grown on the experimental farm of Nanjing Agricultural University in China, 2018. After sowing of 90 days, we sampled fresh leaves from W30 and 082 individual plants to establish parent pools, respectively. For the F_2_ progeny, 100 individuals with contrasting heading trait were selected to establish F_2_ segregation bulks (50 plants with loose leaf, 50 plants with leafy head). Each sample was divided into two sets: one for BSA-Seq, the other for BSR-Seq.

For real time PCR analysis, the male parent (W30) and female parent (082) were sampled, three individual plants were also selected from F_2_ progeny including the extremely heading type plant (HC), non-heading type plant (LT) and another plant with intermediate heading trait (MT). The heading quality order of five individual plants: W30 > HC > MT > LT > 082. The five individual plants were sampled at the apical (a), middle (b) and basal (c) of the soft leaf with three biological replicates (Figure S5). All leaf samples were flash frozen in liquid nitrogen and stored at −80 °C.

### 4.2. Methods of Bulked-Segregant Analysis

#### 4.2.1. The Construction of Illumina Library of BSA-Seq

For bulked segregant analysis (BSA-Seq), four DNA pools were constructed with two parent bulks and two F_2_ segregation bulks. The parent bulks R01 and R02 were constructed with male parent (W30) and female parent (082), respectively. The DNA bulk R03 was constructed by equally mixing the genomic DNAs of 50 outstanding F_2_ individual plants with leafy head, and the R04 bulk was similarly constructed with 50 F_2_ individual plants showing an extremely non-heading trait. The four sequencing libraries were prepared according to the standard protocol of Illumina, then it was sequenced on Illumina HiSeq™2500 platform (Illumina, San Diego, CA, USA).

#### 4.2.2. Alignment with the Reference Genome and Variant Calling

The original files generated from high-throughput sequencing were converted to raw reads by using base calling software Illunima Casava 1.8, the quality evaluation was performed to obtain clean reads as described by Ren et al. (2019) [51]. Then, the filtered short reads were aligned to the reference Chinese cabbage genome (http://brassicadb.org/brad/) using the BWA (Burrows–Wheeler–Aligner) software [52]. SAMtools was used to remove duplication and mask the effects of PCR duplication [53], followed by preprocessing of local realignment and base recalibration using GATK (Genome Analysis Toolkit) [15], and the variant calling of SNPs and small indels were excavated. Finally, the SnpEff software was performed for the annotation of SNPs and small indels [54].

#### 4.2.3. Mapping of Candidate Genomic Regions by Association Analysis

The heterozygous and inconsistent SNPs and small indels (coverage depth > 5×) between two F_2_ contrasting pools (R03 and R04) were selected for calculating the Δ(SNP-index) values, and sliding-window analysis was performed for the association analysis. The regression fitting of LOESS was performed for Δ(SNP-index) on the same chromosome to obtain the associated threshold. The candidate genomic regions were identified with an average *p*-value ≤ 0.01.

### 4.3. Bulked Segregant RNA-Seq Analysis

#### 4.3.1. cDNA Library Preparation of BSR-Seq

For bulked segregant RNA-Seq (BSR-Seq) analysis, four cDNA pools were constructed with two parent bulks (T01 and T02) and two F_2_ segregation bulks (T03 and T04); total RNA was isolated from fresh leaves according to the standard protocol of Illumina. To ensure that a qualified sample was used for the construction of the sequencing library, the RNA concentration was detected using Qubit^®^ 2.0 Fluorometer (Life Technologies, Carlsbad, CA, USA), while the RNA integrity was measured on Agilent 2100 System. The mRNA was enriched and fragmented as described by Gu et al. (2017) [8]. The first strand cDNAs were synthesized by using the shorted mRNA as PCR templates, and the second cDNA strand was synthesized after adding buffer, dNTPs, RNase H and DNA polymerase I; the cDNA was purified by using AMPure XP beads. Then purified double-stranded cDNA was subjected to end-repair and sequenced on the Illumina HiSeq™ 2500 platform (Illumina, San Diego, CA, USA).

#### 4.3.2. Analysis of BSR-Seq Data

The clean reads were obtained by removing the lower reads and adaptors of raw reads. Filtered reads were mapped to the reference Chinese cabbage genome (http://brassicadb.org/brad/) using HISAT2 (v2.0.5). The gene expression level was assessed by using FPKM (Cuffdiff, Fragments Per Kilobase of transcript per Million fragments mapped) method. The differentially expressed genes (DEGs) between the two set pairs of BSR-Seq bulks (T01 vs. T02, T03 vs. T04) were analyzed by using the edgeR software [55], the dispersion was set as 0.1. The false discovery rate (FDR) was obtained through adjusting the hypothesis significance *p*-values with the Benjamini and Hochberg method (1995) [56]. 

The fold change indicated the ratio of expression levels of the same gene between the two groups. Standards for screening differentially-expressed genes: |log_2_ (Fold Change)| ≥ 1, FDR < 0.05. The SNPs and small indels were identified by using the GATK toolkit, sliding-window analysis was performed for Δ(SNP-index) association analysis like BSA-Seq analysis, and all DEGs and genes involved in candidate regions were annotated to GO, COG, KOG, KEGG public databases.

### 4.4. Real-Time Quantitative PCR for Validation

Total RNA was extracted from the frozen leaves by using an RNA extraction kit (Tiangen Biotech, Beijing, China). For each sample, 1 µg of total RNA was used to synthesize the first-strand cDNA by using PrimeScript ™ RT reagent Kit with gDNA Eraser (Takara, Dalian, China). The real time PCR assay was performed with 2μL of diluted cDNA and 10μL of SYBR^®^ Premix ExTaq (Takara, Dalian, China) by using the Applied Biosystems QuantStudio 6 (Thermo Fisher Scientific, Waltham, MA, USA). The amplification program was carried out as follows: 95 °C for 30 s, followed by 40 cycles of 95 °C for 5 s and 60 °C for 30 s. The Chinese cabbage *actin* gene (*Bra028615*) was served as the internal reference. The 2^−ΔΔCT^ method was used to calculate the relative expression levels of special gene between three different parts of mature leaves. The expression analysis was performed in triplicate. The specific primer pairs used in this study are listed in Table 5.

## Figures and Tables

**Table 1:** Summary of sequencing data and the alignment result of BSA-Seq.

Bulk	Clean Reads	Date Generated (Gb)	Q30 (%)	Genome Coverage10× (%)	Average Depth (×)	SNP Number	Alignment Efficiency (%)
R01	37948696	11384608800	92.89	93.43	27.5384	1693338	97
R02	36842550	11052765000	93.55	92.44	26.8289	1663130	97.26
R03	107061946	32118583800	93.10	97.51	74.5499	1671014	96.71
R04	105760141	31728042300	92.81	97.69	75.1521	1744548	97.02

**Table 2:** Candidate genomic regions identified by association analysis of BSA-Seq.

Method	Chromosome	Start	End	Size(M)	Genes
SNP-index	Scaffold000193	62295	162295	0.1	5
small indels-index	A05	10696712	10796712	0.1	13
small indels-index	Scaffold000111	57736	157736	0.1	12
small indels-index	Scaffold000167	153224	253224	0.1	10

**Table 3:** Summary of sequencing data and the alignment result of BSR-Seq.

Bulk	Clean Reads	Date Generated (Gb)	Q30 (%)	SNP Number	Alignment Efficiency (%)
T01	21748815	6524644500	92.63	252616	87.56
T02	17800431	5340129300	92.70	187276	89.90
T03	62663675	18799102500	91.87	285539	88.27
T04	52154937	15646481100	92.45	292860	88.98

**Table 4:** Candidate genomic regions identified by association analysis of BSR-Seq.

Method	Chromosome	Start	End	Size(M)	Genes
SNP-index	A02	18137278	21860151	3.72	401
SNP-index	Scaffold000111	134474	596970	0.46	45
SNP-index	Scaffold000167	11168	12357	0.01	1
SNP-index	Scaffold000262	0	45603	0.05	2
SNP-index	Scaffold000302	27795	28067	0.00	1
SNP-index	Scaffold000317	0	27038	0.03	6
small indels-index	A02	34693040	35853039	1.31	141
small indels-index	Scaffold000111	399069	499069	0.10	10

**Table 5:** Sequences of the specific primers used in RT-qPCR.

Gene Name	Sense Primer	Anti-Sense Primer
*BrYUCCA5*	GTGTGTTCAGTCCGCCCGTTA	ATTCCATCTCTGACCCGGAAACG
*BrGH3.5*	TACGAGCTTGTTGTCACCACTT	TGCGTTCTTTACCGCGTTCT
*BrCBL9*	TCCACGGCTGCCAGAGAGTT	GCCATCGTCAACAACTGAGCTGC
*BrAct*	TTGCTATTCAGGCTGTTCT	CACCATCACCAGAGTCAA
*BrSAUR46*	CCACTCTTCAAGGCACTGCTGGA	GGACAACGTCGAGGAAGGTGCT
*BrMYB1*	AGCTCCCGTAAAGCACCATC	TGTAGTTGCGGTTGTGTGGT
*BrIAA30*	CTCGGACTCAGCTTCGGCTC	CATGATCATTTCTTGATCAGCAGCC
*BrMYB51*	ACCTCCCCGAGATTCCAGAG	ATCCCGGCTTCTCGTTGTTA
*BrGH3.5*	GCCGCAAGAATGTGGTGTTG	CATCGAATGGGACGAGGTGT

## Supplementary Materials

The following are available online at https://www.mdpi.com/2223-7747/8/12/603/s1, Figure S1: KOG function classification analysis of candidate genes identified by the association analysis of BSA-Seq, Figure S2: Gene Ontology (GO) enrichment analysis of differentially expressed genes (DEGs) between two set pairs of cDNA sequencing bulks of BSR-Seq analysis. All genes and the DEGs were assigned to the three GO categories: biological processes, cell components, and molecular functions. (A) T01 vs. T02. (B) T03 vs. T04, Figure S3: KOG function classification analysis of differentially expression genes (DEGs) between four cDNA sequencing bulks. (A) T01 VS. T02. (B) T03 VS. T04, Figure S4: KOG function classification analysis of candidate genes identified by the association analysis of BSR-Seq, Figure S5: Three sampling locations for mature leaves, Table S1: Difference expression genes assigned 5 plant signaling pathways; Table S2: Sequencing results of specific gene.
