# Supplementary material for: Conjunctive Analyses of BSA-Seq and BSR-Seq to Reveal the Molecular Pathway of Leafy Head Formation in Chinese Cabbage
Source: Plants (Basel). 2019 Dec 13;8(12):603. doi: 10.3390/plants8120603 (PMC6963953; doi:10.3390/plants8120603)
Supplement: Supplementary file 1 [file plants-08-00603-s001.zip › Supplementary figure.pdf]

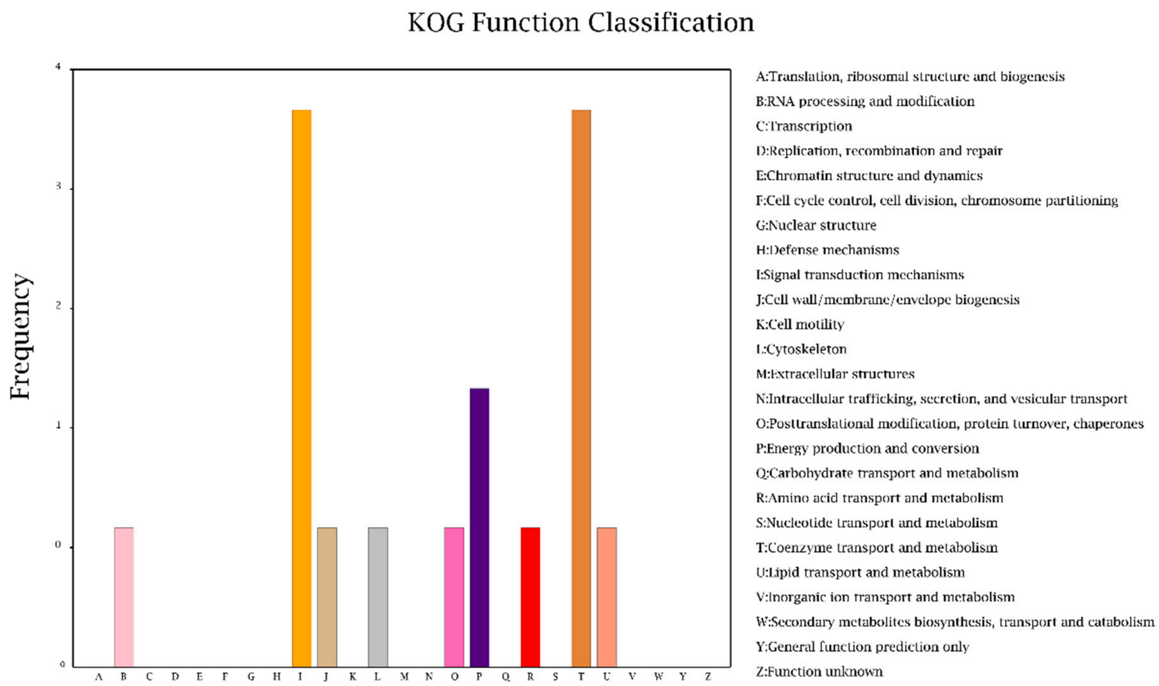

Figure S1. KOG function classification analysis of candidate genes identified by the association analysis of BSA-Seq.

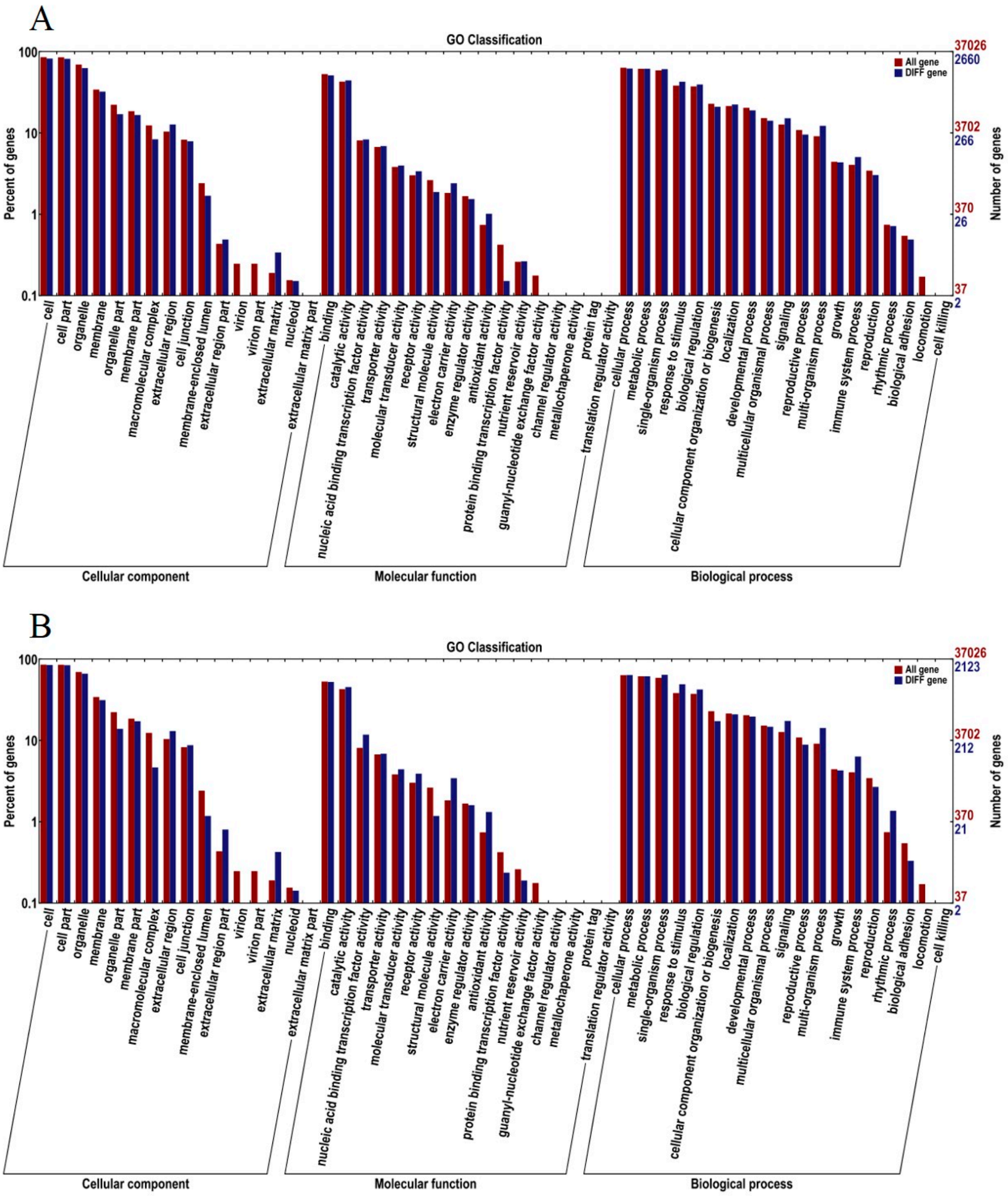

Figure S2. Gene Ontology (GO) enrichment analysis of differentially expressed genes (DEGs) between two set pairs of cDNA sequencing bulks of BSR-Seq analysis. All genes and the DEGs were assigned to the three GO categories: biological processes, cell components, and molecular functions. (**A**) T01 vs. T02. (**B**) T03 vs. T04.

A

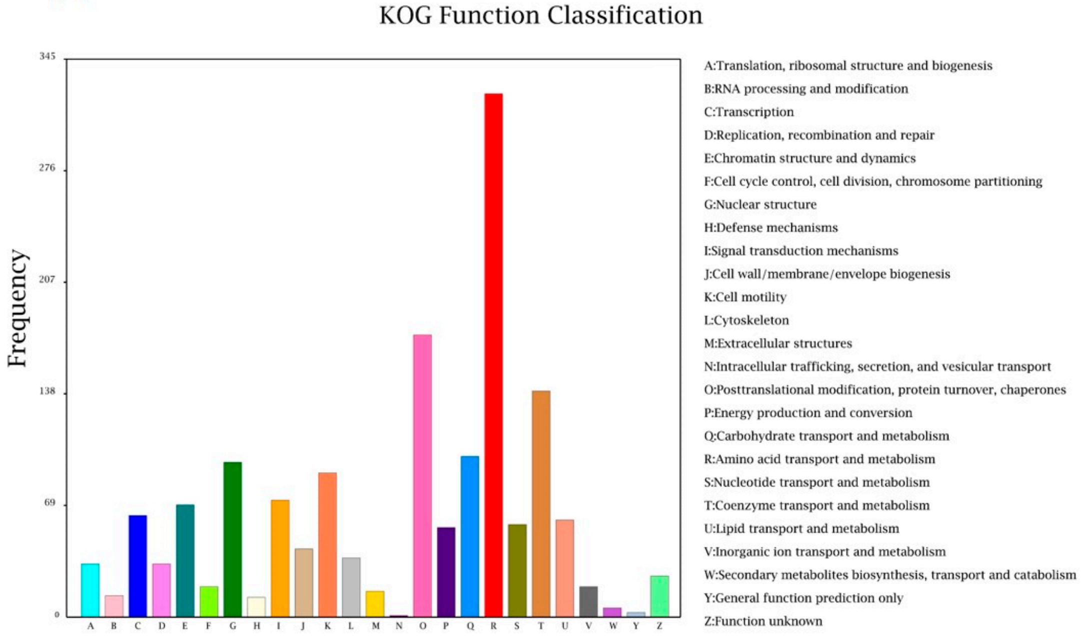

B

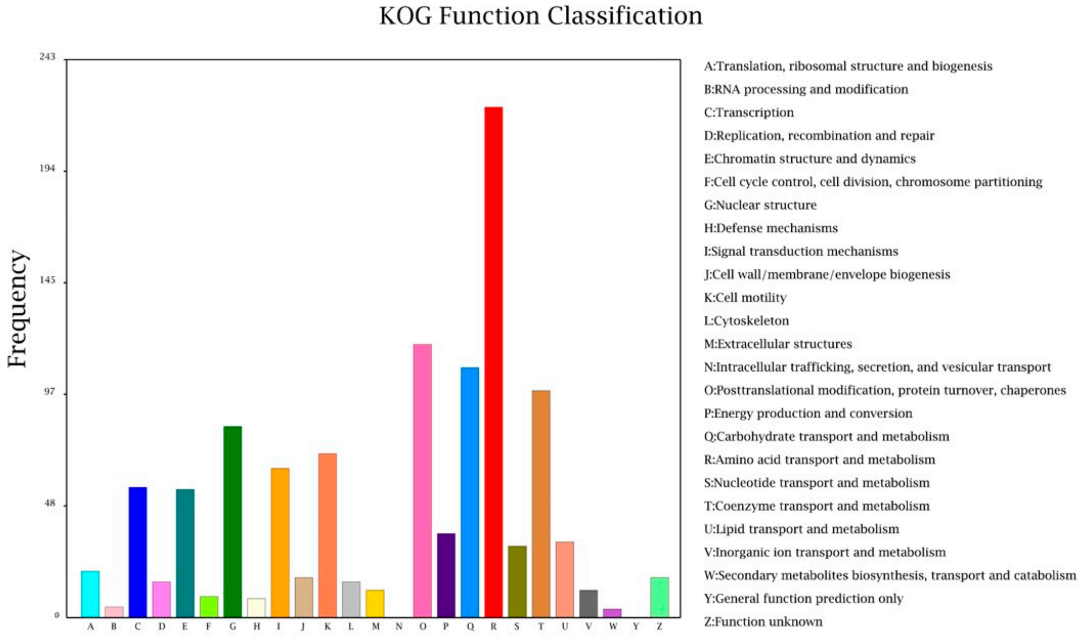

Figure S3. KOG function classification analysis of differentially expression genes (DEGs) between four cDNA sequencing bulks. (**A**) T01 VS. T02. (**B**) T03 VS. T04.

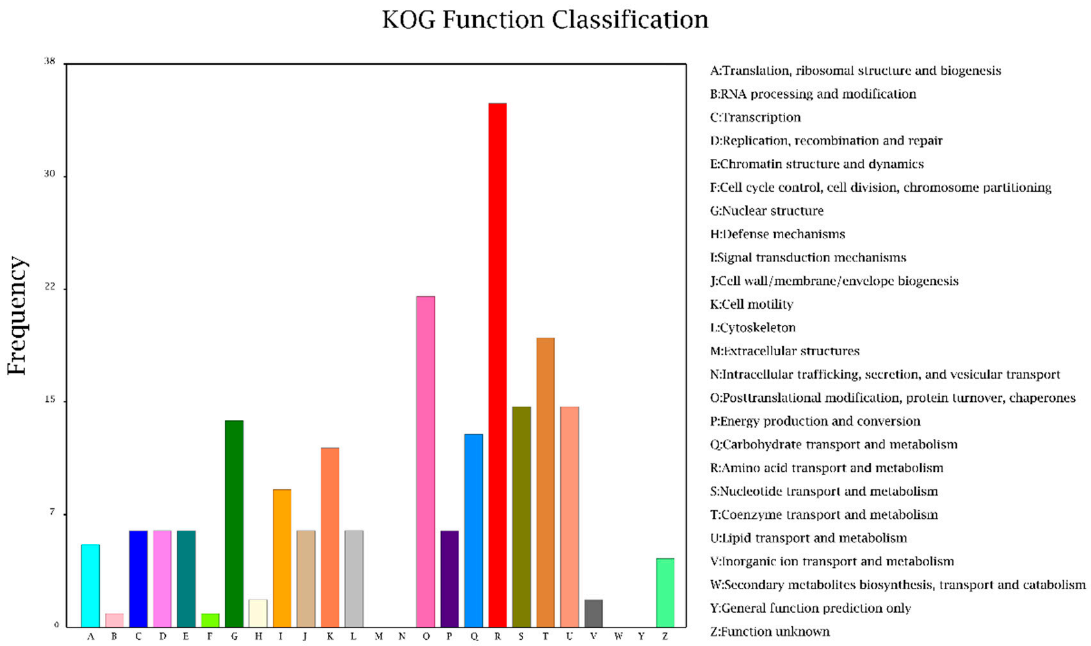

Figure S4. KOG function classification analysis of candidate genes identified by the association analysis of BSR-Seq.

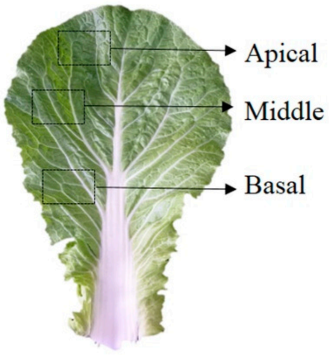

Figure S5. Three sampling locations for mature leaves.
